# Distinct outcomes, ABL1 mutation profile, and transcriptome features between p190 and p210 transcripts in adult Philadelphia-positive acute lymphoblastic leukemia in the TKI era

**DOI:** 10.1186/s40164-022-00265-2

**Published:** 2022-03-11

**Authors:** Ting Shi, Mixue Xie, Li Chen, Wei Yuan, Yungui Wang, Xin Huang, Wanzhuo Xie, Haitao Meng, Yinjun Lou, Wenjuan Yu, Hongyan Tong, Xiujin Ye, Jinyan Huang, Jie Jin, Honghu Zhu

**Affiliations:** 1 Department of Hematology, The First Affiliated Hospital, College of Medicine, Zhejiang University, #79 Qingchun Road, Hangzhou, 310003 Zhejiang Province China; 2 Zhejiang Province Key Laboratory of Hematology Oncology Diagnosis and Treatment, Hangzhou, China; 3 Zhejiang University Cancer Center, Zhejiang University, Hangzhou, China; 4 Zhejiang Laboratory for Systems & Precision Medicine, Zhejiang University Medical Center, Hangzhou, China; 5 Bio-Med Big Data Center, The First Affiliated Hospital, College of Medicine, Zhejiang University, Hangzhou, Zhejiang China; 6 Zhejiang Provincial Key Laboratory of Pancreatic Disease, The First Affiliated Hospital, College of Medicine, Zhejiang University, Hangzhou, Zhejiang China; 7 Program in Clinical Medicine, School of Medicine of Zhejiang University, Hangzhou, Zhejiang China; 8 Department of Physiology, Medical College of Three Gorges University, Yichang, Hubei China

**Keywords:** Ph^+^ ALL, BCR-ABL1, p190, p210, TKIs, scRNA-seq

## Abstract

**Background:**

The differential signaling and outcome of patients with p190 or p210 transcripts of BCR-ABL1 have been systematically investigated in chronic myeloid leukemia rather than in Philadelphia chromosome-positive acute lymphoblastic leukemia (Ph^+^ ALL).

**Methods:**

We analyzed the outcomes and ABL1 mutation profiles in 305 consecutive adult patients with Ph^+^ ALL treated with chemotherapy plus tyrosine kinase inhibitors. We also studied transcriptome features in two newly diagnosed patients with p190 and p210 using single-cell RNA sequencing (scRNA-seq).

**Results:**

P190 and p210 were found in 199 (65%) and 106 (35%) patients, respectively. Compared to patients with p190, a higher white blood cell count (*p* = 0.05), platelet count (*p* = 0.047), BCR-ABL1 transcript level (*p* < *0.001*), and lower bone marrow blasts (*p* = 0.003) were found in patients with p210. Patients with p210 had fewer types of ABL1 mutations (4 vs. 16) and a higher prevalence of T315I and E225K/V mutations (91.3% vs. 68.6%; *p* = 0.031). Patients with p210 had a similar complete remission rate (91.0% vs*.* 90.1%; *p* = 0.805) but a lower complete molecular remission rate at 1 month (9.9% vs. 22.0%; *p* = 0.031) compared with p190. Patients with p210 had lower 3-year overall survival (OS) and disease-free survival (DFS) rates than those with p190 (3-year DFS: 10.4% vs. 9.2%, *p* = 0.069, 3-year OS: 44.3% vs. 38.2%, *p* = 0.018, respectively). Multivariate analysis revealed that p210 was independently associated with worse OS [HR 1.692 (95% CI 1.009–2.838), *p* = 0.046]. Allogeneic hematopoietic stem-cell transplantation (allo-HSCT) was associated with a better prognosis in patients with p210 (*p* < 0.0001). In addition, scRNA-seq data showed distinct molecular and cellular heterogeneity between bone marrow cells of the two transcripts.

**Conclusions:**

Ph^+^ ALL patients with p190 and p210 had different clinical characteristics, outcomes, ABL1 mutation profiles, and transcriptome features. Allo-HSCT could improve the outcomes of patients with p210.

**Supplementary Information:**

The online version contains supplementary material available at 10.1186/s40164-022-00265-2.

## Background

Acute lymphoblastic leukemia (ALL) is a commom hematologic disorders and represents a significant global health burden. A significant increase in the global incidence of ALL has been reported from 1990 to 2017 [1, 2]. The Philadelphia chromosome (Ph), resulting from the translocation between chromosomes 9 and 22 [t(9;22)(q34;q11.2)], is an well-known cytogenetic abnormality in ALL and characterized by the rearrangement of BCR-ABL1 oncogene. P210 and p190 are the most common fusion proteins encoded by BCR-ABL1. The two types of oncoproteins exhibit distinct intrinsic differences, including structure, kinase activity, and signaling pathways [3–7]. Different outcomes between patients with p190 and p210 transcripts have been systematically investigated in chronic myeloid leukemia (CML) [8–11]. However, some studies with small samples have reached inconsistent conclusions in Ph^+^ ALL [12–31]. Therefore, large sample studies are needed to elucidate the prognostic value of p190 and p210 transcripts in Ph^+^ ALL.

Herein, we retrospectively examined the clinical characteristics and outcomes of p190 and p210 transcripts in a large sample of 305 adult patients with Ph^+^ ALL treated with chemotherapy plus tyrosine kinase inhibitors (TKIs). We performed single-cell RNA sequencing (scRNA-seq) analysis on 17,111 bone marrow cells from one p190 sample and one p210 sample to study the potential heterogeneity. Clinically, our results showed distinct outcomes and ABL1 mutation profiles between p190 and p210 transcripts. P210 was an independent predictor of poor survival, and allogeneic hematopoietic stem-cell transplantation (allo-HSCT) could improve patient outcomes. In addition, our scRNA-seq data indicate distinct molecular and cellular heterogeneity between the two transcripts, which adds novel insights into the biology of disease development and paves the way for exploring the key mechanisms of disease treatment.

## Methods

### Patients

Between January 2010 and June 2020, a total of 311 consecutive adult patients with de novo Ph^+^ ALL were reviewed, and none of them had a history of malignant disease involving CML and myeloproliferative diseases (MPD). Six patients were excluded due to a lack of available data on transcripts. The diagnostic criteria were according to the one recommended by the World Health Organization. The study was approved by the Institutional Review Board of the First Affiliated Hospital, Zhejiang University School of Medicine.

### Treatment and response definitions

According to the physical condition, patients were treated with intensive (CALLG2008 protocol or Hyper-CVAD) or dose-reduced chemotherapy regimens such as induction, consolidation, and maintenance therapy [32, 33]. TKIs were recommended to all patients, and the dosage of TKIs was 300–400 mg/d for imatinib, 100–140 mg/d for dasatinib, 600–800 mg/d for nilotinib, 600 mg/d for flumatinib, and 30–45 mg/d for ponatinib. TKI interruption was dependent on the blood cell counts: neutrophils < 1*10^9^/L or platelets < 50*10^9^/L. Prophylaxis of central nervous system leukemia (CNSL) was performed in all patients. All eligible patients were recommended to undergo allo-HSCT when they achieved complete remission (CR).

The definition of CR is the presence of less than 5% blasts in the bone marrow, with ≥ 1*10^9^/L neutrophils and ≥ 100*10^9^/L platelets in the peripheral blood, and the absence of extramedullary disease. Refractory was defined as failure to achieve CR at the end of induction. The methods of BCR-ABL1 minimal residual disease (MRD) monitoring and mutation analysis have been described previously [32]. The major molecular remission (MMR) was defined as BCR-ABL1 transcripts less than 0.1% by quantitative PCR. Complete molecular remission (CMR) was defined as an undetectable BCR-ABL1 transcript level. Relapse was defined by recurrence of > 5% bone marrow blasts or by the presence of extramedullary disease. According to the setting of data analysis, disease-free survival (DFS) was defined as the time from the date of first CR to the date of relapse, transplantation, death, or last follow-up (Dec 31, 2020). Overall survival (OS) was calculated from the date of disease diagnosis until the date of transplantation, death, or last follow-up.

### Analysis of single-cell transcriptomes

We performed scRNA-seq on bone marrow cells obtained from two newly diagnosed patients with p190 and p210 at the 1st Affiliated Hospital of Zhejiang University. ScRNA-seq data processing: scRNA-seq of gene expression was performed using the 10X Genomics Chromium Single Cell platform [34]. Basic analyses, such as quality control, feature detection, mapping, and feature-barcode generation, were performed by the 10× Genomics Cell Ranger 6.0.0 software pipeline. Seurat [35] (V4.0.3) was applied to following analysis. Cells with fewer than 25% of reads mapped to tomitochondrial genes were retained. Cell clustering and dimension reduction were also performed by Seurat [35] (V4.0.3). We used marker genes to identify the normal cells. Combining two strategies for cell type identification, SingleR [36] (V1.4.1) analysis and “FeaturePlot” in Seurat, we finally identified most of the cells as tumor cells. DEGs were identified using the Wilcox method implemented by the “FindMarkers” function in Seurat. Gene functional enrichment analysis was performed using ClusterProfiler [37] (V 4.0.5). To compare the differential expression of p190 tumor cells versus p210 tumor cells, GSEA was performed by GSEA [38, 39] (V4.1.0) software.

### Statistical methods

SPSS 22.0 software was used for the statistical analysis of these data. The chi-square test, t test, or Mann–Whitney U test were used for descriptive statistical analyses. The pearson correlation analyses was conducted to assess correlation. The Kaplan–Meier method was used to estimate DFS and OS, and differences among curves were evaluated by the log-rank test. A Cox regression model was used to identify prognostic variables in patients. Only variables with p < 0.20 in the univariate analyses were included in the multivariate model; backward elimination was used until all variables showed a p value of < 0.05. A value of p < 0.05 was considered statistically significant.

## Results

### Baseline characteristics by transcript type

We identified 305 patients with available breakpoint data, of whom 148 (48.5%) patients were male. The median age was 44 years (range 14–83 years); 15.1% of patients were aged 60 years or older. P190 transcripts was detected in 199 (65%) patients. The baseline characteristics of all patients classified by transcripts are summarized in Table 1. Our results showed that patients with p190 presented with lower white blood cell (WBC) counts (*p* = 0.05), platelet counts (*p* = 0.047), and BCR-ABL1 transcript levels (*p* < 0.001), while patients with p210 presented with lower bone marrow blasts (*p* = 0.003). There were no statistically significant differences in sex, age, hemoglobin, lactate dehydrogenase, ferritin or karyotype between patients with p190 or p210 transcripts. Additionaly, the prognostic impact of some additional chromosomal abnormalities (ACAs) is established, and the presence of −7, + 8, −9/9p, + der(22)t(9;22), hypodiploidy (< 44 chromosomes), and complex karyotype (≥ 5 chromosome abnormalities) constituted a high-risk karyotypes [40]. In our analysis, the proportion of high-risk karyotypes was similar between p190 and p210 (p190 n = 22, 43.1% of the ACAs cohort; p210 n = 19, 16% of the ACAs cohort; *p* = 0.06).

**Table 1:** Characteristics of patients with p190 and p210 in Ph^ + ^ALL

Characteristics	p190 (n = 199)	p210 (n = 106)	*p*-value
Sex (n, M/F)	101/98	47/59	0.286
Age [years, M(range)]	43.0 (14.0–83.0)	46.0 (14.0–71.0)	0.130
WBC [*10E9/L, M(range)]	18.0 (0.2–403.4)	33.7 (1.0–461.9)	0.050
Hb [g/L, $\overline{x}$ ± s]	97.7 ± 28.9	92.3 ± 28.7	0.120
PLT [*10E9/L, M(range)]	30.0 (2.0–460.0)	38.0 (2.0–403.0)	0.047
LDH [U/L, M(range)]	599.5 (134.0–7638.0)	525.5 (128.0–4187.0)	0.385
Ferritin [ng/ml, M(range)]	909.8 (37.3–88,150.0)	874.4 (13.7–10,943.1)	0.771
BM blast [%, M(range)]	86.3 (23.0–98.0)	81.0 (22.0–96.0)	0.003
Relative BCR/ABL quantification [%, M(range)]	44.0 (7.2–165.9)	77.9 (25.0–195.0)	< 0.001
Karyotype			0.843
With ACAs Without ACAs Unknown	51	31	
	94	54	
	54	21	

*WBC* white blood cell, *Hb* hemoglobin, *PLT* Platelet, *LDH* Lactate dehydrogenase, *BM* bone marrow, *ACAs* additional cytogenetic abnormalities, *CNS* central nervous system, *TKIs* tyrosine kinase inhibitors

### Frequency and spectrum of BCR-ABL1 mutations by transcript type

**Fig. 1:**
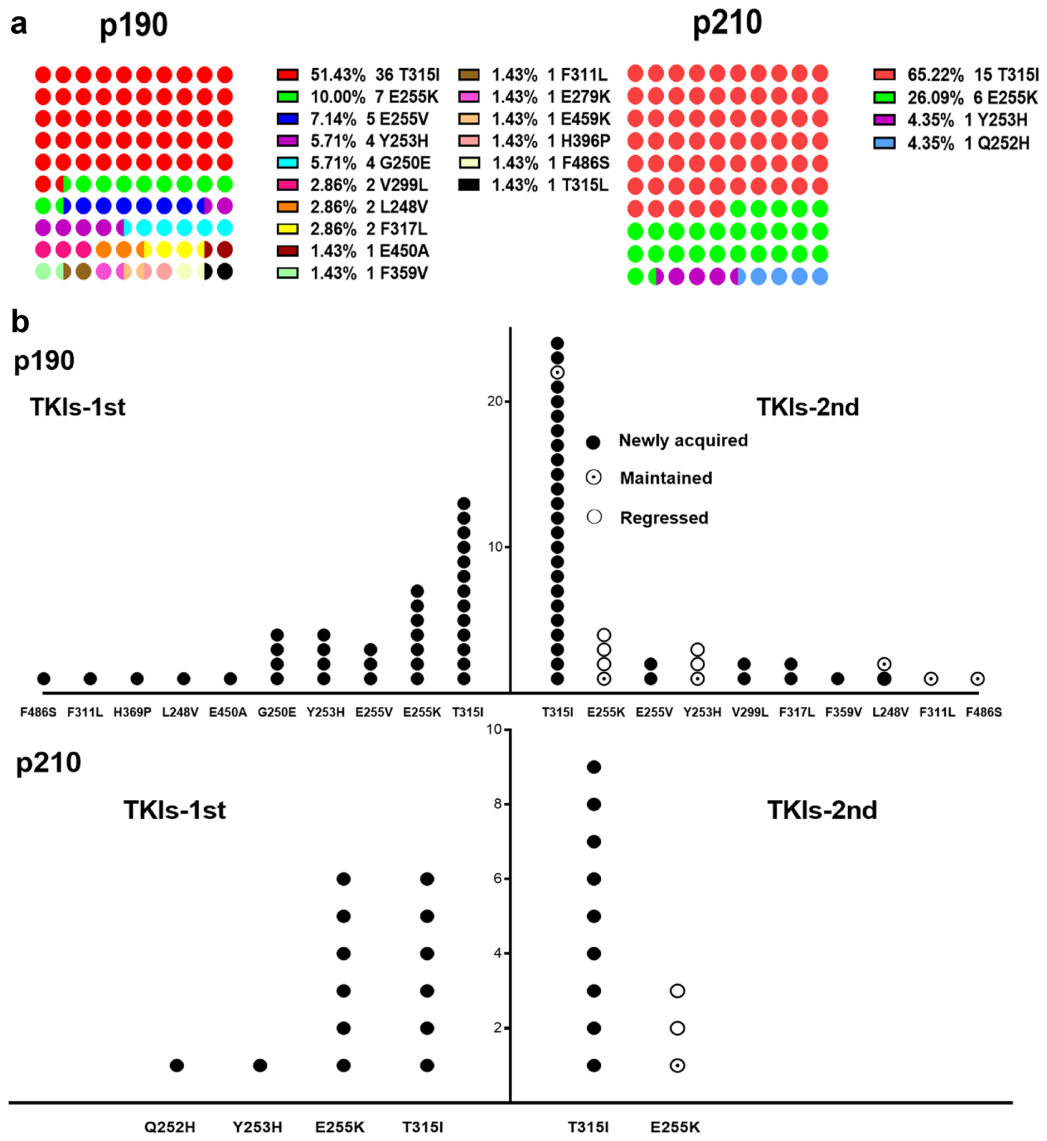
Frequency and spectrum of BCR-ABL1 mutations by transcript type. **a** Localization of BCR-ABL1 kinase domain mutations based on BCR-ABL1 transcripts and **b** detected at the time of imatinib and dasatinib failure

Overall, BCR-ABL1 mutations were identified in a group of 69 patients (20 cases with p210, 49 cases with p190). Most were related to relapse. The spectra of 17 types of amino acid substitutions at 15 different residues were detected, including T315I/L, E255K/V, G250E, Y253H, V299L, L248V, F311L, F317L, F359V, F486S, E450A, E479K, E459K, H396P, and Q252H (Fig. 1a). We observed a wide variety of mutation types in patients with p190, and the mutation types of patients with p210 mainly occupied P-loop (amino acids from 248 to 256) and gatekeeper (T315I) regions. Forty-eight (69.6%) patients (17 cases with p210, 31 cases with p190) had a single mutation; 18 (26.1%) patients (three cases with p210, 15 cases with p190) had two mutations, and three (4.3%) patients with p190 had three mutations (Y253H, T315I, T315L; G250E, E255V, T315I; E255V, F359V, T315I). The frequency of T315I and E255K/V mutations was significantly higher in 210 patients with p210 (91.3% vs. 68.6%; *p* = 0.031). The localization of BCR-ABL1 mutations detected at the time of TKI-1st failure and at the time of TKI-2nd failure is shown in Fig. 1b. E279K and E459K were detected after ponatinib administration.

### Overall treatment results by transcript type

**Fig. 2:**
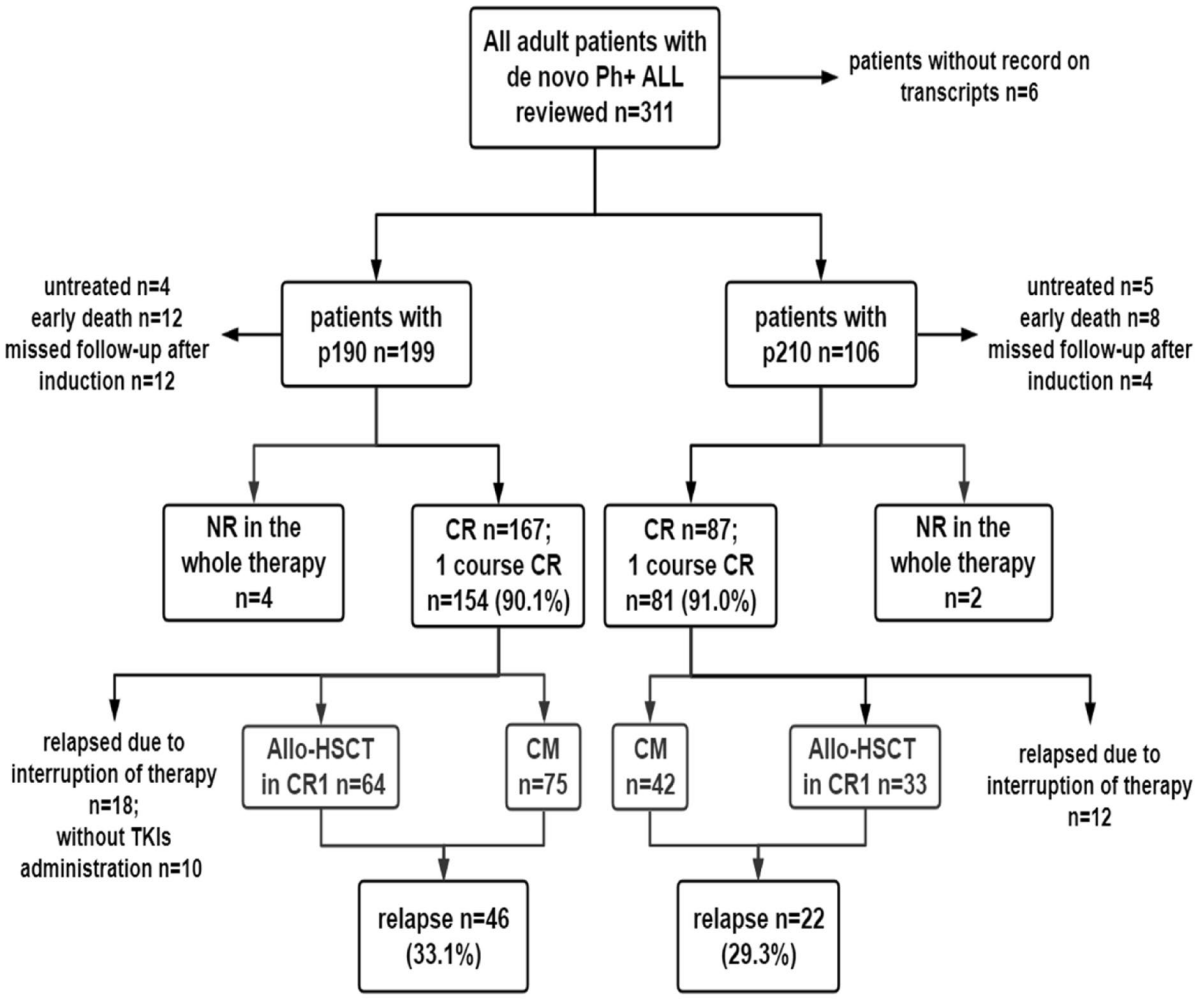
Flow of this retrospectively study

Figure 2 details the study flow. Of those patients included, nine (5 patients with p210, 4 patients with p190) were diagnosed without further treatment, and five patients with p190 died before induction therapy. Early deaths occurred in 15 cases during the first induction therapy (8 cases with p210, 7 cases with p190), and 16 cases failed to be followed up after the first induction therapy (4 cases with p210 group, 12 cases with p190). Afterward, 260 patients out of the 305 Ph^+^ ALL patients were evaluable for therapeutic response. After one course induction therapy, 154 of 171 patients with p190 and 81 of 89 patients with p210 achieved CR. CR rates were similar among patients with p190 or p210 transcripts (90.1% vs. 91.0%, *p* = 0.805). Patients with p190 transcripts had a higher rate of CMR to induction therapy (22% vs. 9.9%; *p* = 0.031) (Table 2). And there was no correlation between BCR-ABL1 transcription level and CMR to induction therapy (r = −0.123; *p* = 0.082).

**Table 2:** Comparison of the response and relapse rate of the p190 and p210 patients

	p190	p210	*p*-value
CR1 rate (%)	90.1% (154/171)	91.0% (81/89)	0.805
CMR rate (%)			
1 month	22% (29/132)	9.9% (7/71)	0.031
3 months	43.2% (54/125)	41.4% (29/70)	0.810
MMR rate (%)			
1 month	37.9% (50/132)	28.2% (20/71)	0.165
3 months	66.4% (83/125)	61.4% (43/70)	0.486
Relapse rate (%)	33.1% (46/139)	29.3% (22/75)	0.573

*CR* complete remission, *CMR* complete molecular remission, *MMR* major molecular remission

For a better comparability of relapse and survival data, we excluded 30 patients who were subjected to relapse due to interruption of treatment (chemotherapy or TKIs). In addition, 10 patients were also excluded due to the absence of documented TKI records. The remaining 220 patients were included for further analysis. The pretreatment characteristics of these patients are summarized in Additional file 2: Table S1. There were no statistically significant differences between the characteristics at the time of diagnosis (including age, WBC, and CNS disease) of patients with p210 and p190. Throughout therapy, two patients with p210 and four patients with p190 did not achieve remission. The relapse rate was 33.1% for patients with p190 versus 29.7% for patients with p210, and no clear statistically significant difference was observed (*p* = 0.573). A group of 111 patients underwent allo-HSCT, and most were from related and haploidentical donors. There were 97 patients who received allo-HSCT in CR1, of whom 33 presented with p210. And the reasons not move to allo-HSCT in CR1 are as follows: donor availability, economic burden and physical condition of the recipient. After a median follow-up of 20.8 months (range 1.0–132.0), the 5-year DFS and OS were 48.3% and 46.2%, respectively.

**Fig. 3:**
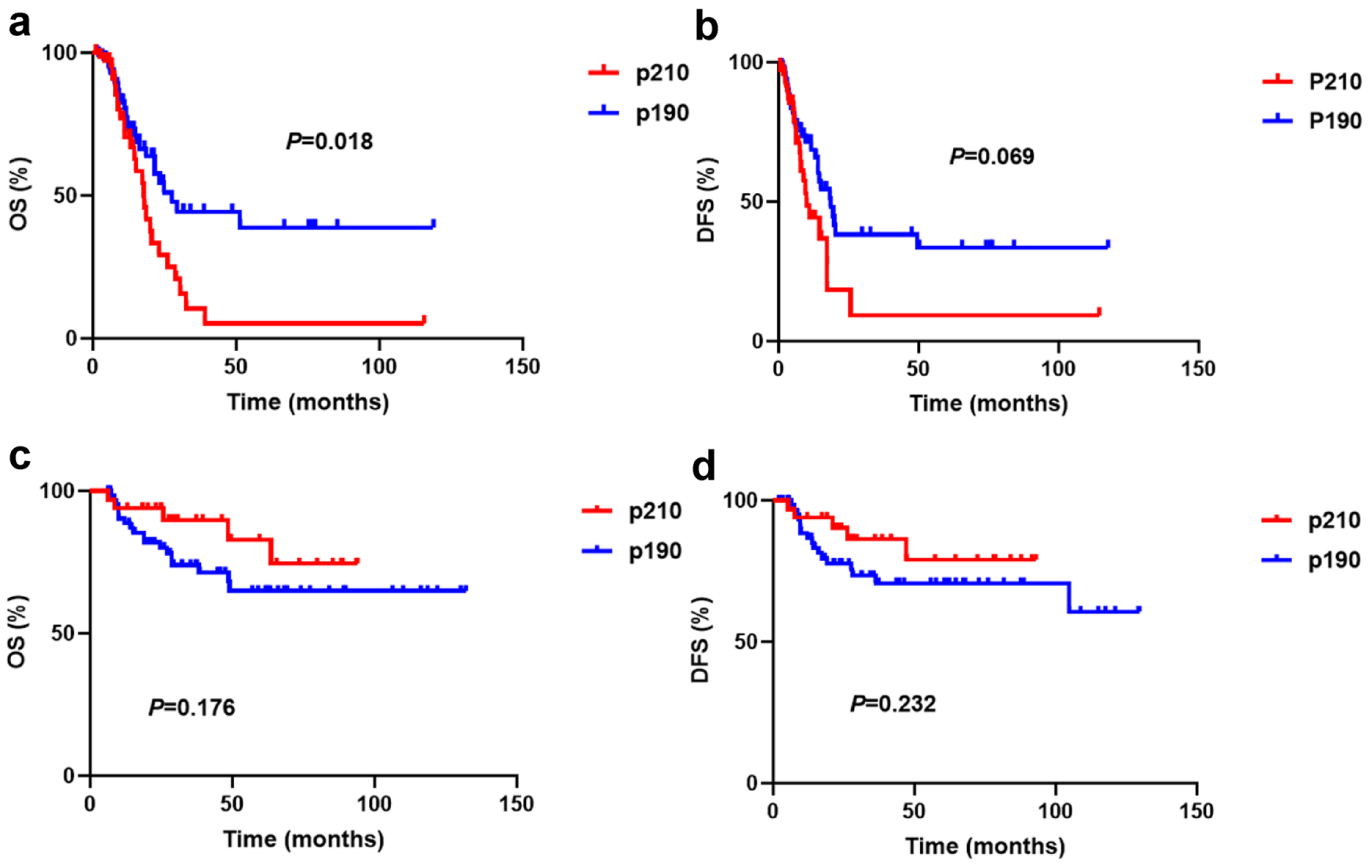
Outcomes for Ph^+^ ALL patients based on BCR-ABL1 transcripts. **a** Overall survival (OS) and **b** Disease-free survival (DFS) in the whole group; **c** OS and **d** DFS in the HSCT subgroup

We then excluded the impact of transplantation in the following analysis: the analysis endpoints of all transplant patients were censored at the time of allo-HSCT. The median follow-up period was 7.6 months (range 1.0–118.8). Overall, the 3-year DFS and OS were 29.4% and 30.8%, respectively. Patients with p190 had a longer survival than those with p210 (3-year DFS: 10.4% vs. 9.2%, *p* = 0.069, 3-year OS: 44.3% vs. 38.2%, *p* = 0.018, respectively) (Fig. 3A and B). We further analyzed the transplant patients in CR1 to see whether the type of BCR-ABL1 transcripts can make different influence on survival of the patients. Our analysis showed that there were no differences in outcomes between patients with p190 and p210, and the median DFS and OS of the two transcripts were undefined (*p* = 0.232 and *p* = 0.176, respectively) (Fig. 3C and D). Definitely, allo-HSCT could significantly improve the prognosis of patients with p210. (*p* < 0.0001) (Additional file 1: Figure S1).

Factors influencing OS and DFS in adult patients with Ph^+^ ALL were analyzed by univariate and multivariate Cox regression analyses, as shown in Table 3 and Additional file 2: Table S2. We found that p210 was the only independent risk factor for OS in adult patients with Ph^+^ ALL (hazard ratio (HR): 1.692; 95% confidence interval (CI): 1.009–2.838; *p* = 0.046). The DFS was not significantly affected (HR = 1.543, 95% CI 0.942–2.525; *p* = 0.085).

**Table 3:** Risk factors for overall survival in univariate and multivariate analysis

Variable	Univariate analysis			Multivariate analysis		
	HR	95% CI	p-value	HR	95% CI	p-value
Age [≥35/ <35 (years)]	1.077	0.553–2.100	0.827	–	–	–
WBC count [≥ 30/ < 30 (*10E9/L)]	1.949	1.162–3.269	0.011	1.823	1.082–3.072	0.024
PLT count [<30/ ≥30 (*10E9/L)]	1.154	0.690–1.932	0.585	–	–	–
Cytogenetics (with ACAs/without ACAs)	0.966	0.540–1.729	0.909	–	–	–
BCR–ABL1 transcript (p210/p190)	1.833	1.099–3.057	0.020	1.692	1.082–2.838	0.046
Initial TKIs administrating (2nd/1st)	0.675	0.331–1.376	0.279	–	–	–
CNS involvement (yes/no)	1.284	0.546–3.022	0.567	–	–	–

*WBC* white blood cell, *PLT* platelet, *ACAs* additional cytogenetic abnormalities, *CNS* central nervous system, *TKIs* tyrosine kinase inhibitors, *HR* hazard ratio, *CI* confidence interval

### ScRNA-Seq depicts the distinct cell types and gene expression between patients with p190 and p210

After quality control, patients with p190 and p210 captured a total of 8336 and 8775 cells, respectively. After dimensionality reduction and unsupervised cell clustering, five clusters (tumor cells, T-cells, B-cells, monocytes, and bone marrow and progenitor cells) were well classified (Additional file 1: Figure S2a). The expression of marker genes indicated T cells (CD30), B cells (MS4A1), monocytes (S100A8) and bone marrow and progenitor (HBB) (Additional file 1: Figure S2b)). The enrichment of T cells, monocytes and bone marrow and progenitor cells was confirmed as the predominant constituent in patients with p210 (Additional file 1: Figure S2c). Consistent with the clinical features we described above, the number of tumor cells in p210 patients was lower than that in p190 patients. We identified the top 6 upregulated and downregulated differentially expressed genes of the two samples (Additional file 1: Figure S2d). To obtain a broad understanding of enriched gene signatures in p190 and p210 transcripts, we performed GO (Gene Ontology) and KEGG (Kyoto Encyclopedia of Genes and Genomes) enrichment analyses. The p190 sample had strong signatures of interferon gamma responses, antigen processing and presentation (Additional file 1: Figure S2e–h). Together, these results demonstrate distinct molecular and cellular heterogeneity between the bone marrow cells of the two transcripts.

## Discussion

In the present study, we found that Ph^+^ ALL patients with p190 and p210 transcripts have distinct clinical characteristics, outcomes, ABL1 mutation profiles, and transcriptome features. P210 was an independent predictor of poor survival, and allo-HSCT improved patient outcomes. This study adds novel insights into risk-stratifying and individualized treatment for adult patients with Ph^+^ ALL.

It has been demonstrated that p190 and p210 are associated with different clinical characteristics and outcomes in CML, and p190 is typically recognized with inferior outcomes [8–11]. However, no consensus has been reached on the different BCR-ABL1 transcripts in Ph^+^ ALL, either in clinical characteristics or outcome, which may be related to the following three reasons: (1) the relatively small sample sizes; (2) the heterogeneity of the research objects, which included patients with Ph^+^ acute myeloid leukemia, acute undifferentiated leukemia, a preceding antecedent of CML, or pediatric population; and (3) the inconsistency in chemotherapy protocols, proportion of allo-HSCT and use of TKIs. Our study is the largest monocentric study reported thus far, aiming to systematically evaluate the prognostic value of BCR-ABL1 transcripts in adult patients with Ph^+^ ALL treated with relatively uniform regimens.

The incidence of p190 in our adult population was 65%, similar to that range reported by others [12–19]. Our analysis showed that patients with p210 were more likely to have a higher WBC, platelet count, relative BCR-ABL1 quantification, and lower bone marrow blasts than those with p190. These findings echo the work of others [13, 14, 20]. Furthermore, we observed a significant difference in early molecular response between the two transcripts rather than later response. A similar finding was reported by the GIMEMA group in adult Ph^+^ ALL patients treated with dasatinib plus steroids [15]. They indicated that patients with p190 showed a more rapid molecular response than p210 patients, with the difference most apparent on Days 22 and 43. In addition, this is in accordance with a retrospective study aiming to compare the efficacy and safety of TKI-1st and TKI-2nd in front-line treatment. Although p190 is a higher carrier in patients receiving TKI-1st, they still reported a higher early molecular response in patients with p190 [21]. Recently, data from the MDACC group suggested that the cumulative MMR and CMR rates before relapse or allo-HSCT were similar between the different BCR-ABL1 transcripts [19]. It is conceivable that the two transcripts are indeed similar in the later molecular response. In contrast, we noted that the CR rate after induction therapy and relapse rate did not differ significantly between the two groups of p190 and p210, which agrees with previous findings [12, 13]. These observations indicate that patients with different transcripts have a heterogeneous sensitivity to induction therapy in terms of molecular response.

As noted above, the prognosis differences are clearly influenced by the patients' characteristics and therapy protocols. Correspondingly, we ruled out the impact of transplantation. We observed that outcome was significantly affected by the type of BCR-ABL1 transcript in the analysis of the whole group. Moreover, in our series, p210 was an independent prognostic factor conferring inferior OS. We analyzed the possible reasons for the worse outcome of patients with p210. First, it has been indicated that the cell of origin of the p210 clone took place in a lymphomyeloid hematopoietic stem cell, while the p190 clone took place in committed B-cell progenitors [41]. Jones et al. described p210-ALL as a disease with a CML background, which is characterized by higher BCR-ABL1 transcript levels and more frequent persistent BCR-ABL1 expression [20]. This phenomenon may be the reason for the inferior prognosis of p210 disease. Second, it has been suggested that P-loop and gatekeeper mutations in the BCR-ABL1 kinase domain have worse prognosis than nonP-loop mutations [42, 43]. To the best of our knowledge, there have been no data regarding the distribution of mutation types between p190 and p210 transcripts. As shown in our data, the frequency of T315I and E255K/V mutations was significantly higher in patients with p210. Conceivably, such poor outcomes of patients with p210 might be induced by the relative preference for developing such mutations; a larger sample size is needed.

Third, our scRNA-seq data of bone marrow samples from patients with p190 and p210 showed distinct molecular and cellular heterogeneity. Similarily, Xiao, et al. found that the JNK exerts different effect in the maintenance of Ph^+^ ALL cells and CML cells [44]. This may lead to different responses of the two diseases to therapy. More importantly, we believed that this issue is worth to explore with more cases, and we are on our way. As the management of Ph^+^ ALL is continuously evolving, the prognosis has improved significantly. Of particular note is the immunotherapies, which exerts great promise in the treatment of Ph^+^ ALL [45, 46]. The application of more potent drugs or combined therapy may have weakened the difference in prognosis between p190 and p210 to some extent. In our analysis, the majority of patients were treated with imatinib (80%, 175/220). Due to the relatively small number of patients with p210 studied, we could not perform survival analysis between the two transcripts in the setting of TKIs-2nd. Of note, the results from other studies performed on dasatinib, blinatumomab, and allo-HSCT showed no difference in prognosis between p190 and p210 [22, 27, 28, 31]. These results suggest that the adverse prognosis of p210 can be offset by a highly potent therapy.

There are some limitations in our study. Next-generation sequencing was not routinely performed at diagnosis. The role of other mutations, such as IKZF1 CDKN2A/B deletions, which have been demonstrated to be important prognostic factors, could not be assessed in this study. Moreover, fewer patients received second-generation TKIs, and the relatively short follow-up time prevented us from investigating in detail. A small number of patients with scRNA-seq is hardly represent the features of patients with p190 or p210. Given the retrospective nature of this study, further investigation is warranted to confirm these findings through larger prospective studies.

## Conclusion

We found that Ph^+^ ALL patients with p190 and p210 transcripts have different clinical characteristics, ABL1 mutation profiles, outcomes, and transcriptome features. More aggressive treatment, such as allo-HSCT, may improve the outcomes of patients with p210.

## Supplementary Information

**Additional file 1: Figure. S1** Outcomes for patients with p210 stratified by treatment protocols (**A**) Overall survival (OS) and (**B**) Disease-free survival (DFS). Figure. S2 4 Single-cell RNA-seq analysis. **A,** tSNE plot of 17,111 cells, colored by the origin samples (top) and cell types (bottom). **B**, Expression of marker genes for the normal cell types defined above each panel. **C**, Barplot of ratios of cells in each cell type. **D**, Violin plots of top 6 upregulated and downregulated differentially expressed genes between two samples among Tumor cells. **E**, Dotplot of top 10 enriched GO terms of Tumor cells. Enrichment analysis used significantly differentially expressed genes (|log2FC| > 1.5, p value < 0.05 ) between two samples. **F**, Dotplot of top 10 enriched KEGG pathways of Tumor cells. Enrichment analysis used significantly differentially expressed genes (|log2FC| > 1.5, p value < 0.05 ) between two samples. **G**, Differences in GO scored by GSEA between b190 and b210 samples’ Tumor cells. **H**, Differences in KEGG pathways scored by GSEA between b190 and b210 samples’ Tumor cells.

**Additional file 2: Table S1.** Characteristics of patients included in for relapse and survival analysis. **Table S2.** Risk Factors for Disease-free survival (DFS) in Univariate and Multivariate Analysis

## Data Availability

All data will become publicly available upon request from the corresponding authors.

## Abbreviations

Ph^+^ ALL: Philadelphia chromosome-positive acute lymphoblastic leukemia
CML: Chronic myeloid leukemia
MPD: Myeloproliferative diseases
TKIs: Tyrosine kinase inhibitors
scRN: A-seq single-cell RNA sequencing
allo-HSCT: Allogeneic hematopoietic stem-cell transplantation
CNSL: Central nervous system leukemia
CR: Complete remission
MRD: Minimal residual disease
MMR: Major molecular remission
CMR: Complete molecular remission
DFS: Disease-free survival
OS: Overall survival
WBC: White blood cell
HR: Hazard ratio
CI: Confidence interval
GO: Gene Ontology
KEGO: Kyoto Encyclopedia of Genes and Genomes
