# Supplementary figures and images for: Distinct outcomes, ABL1 mutation profile, and transcriptome features between p190 and p210 transcripts in adult Philadelphia-positive acute lymphoblastic leukemia in the TKI era
Source: Exp Hematol Oncol. 2022 Mar 11;11:13. doi: 10.1186/s40164-022-00265-2 (PMC8915539; doi:10.1186/s40164-022-00265-2)

## Slide 1
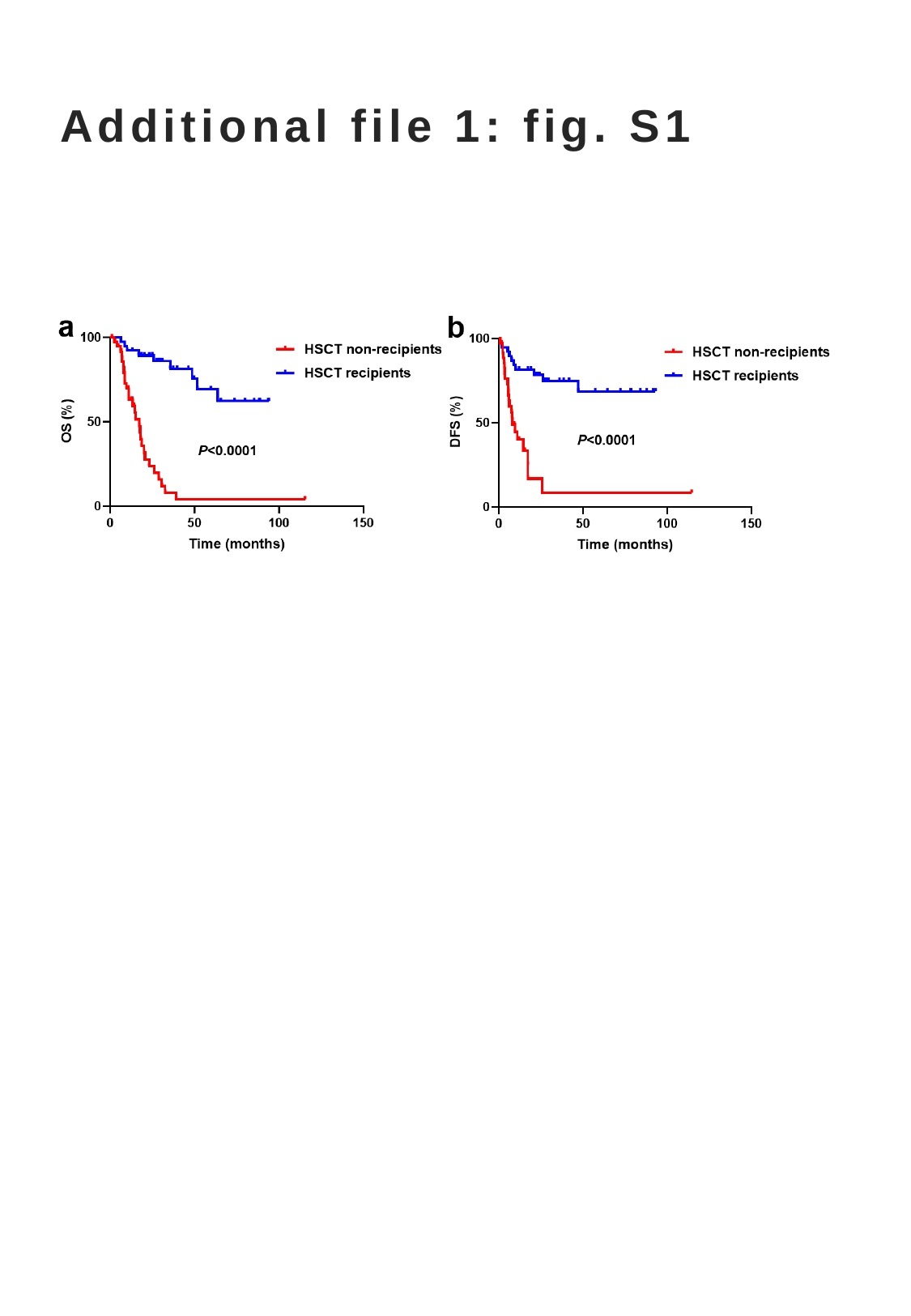

# Additional file 1: fig. S1

## Slide 2
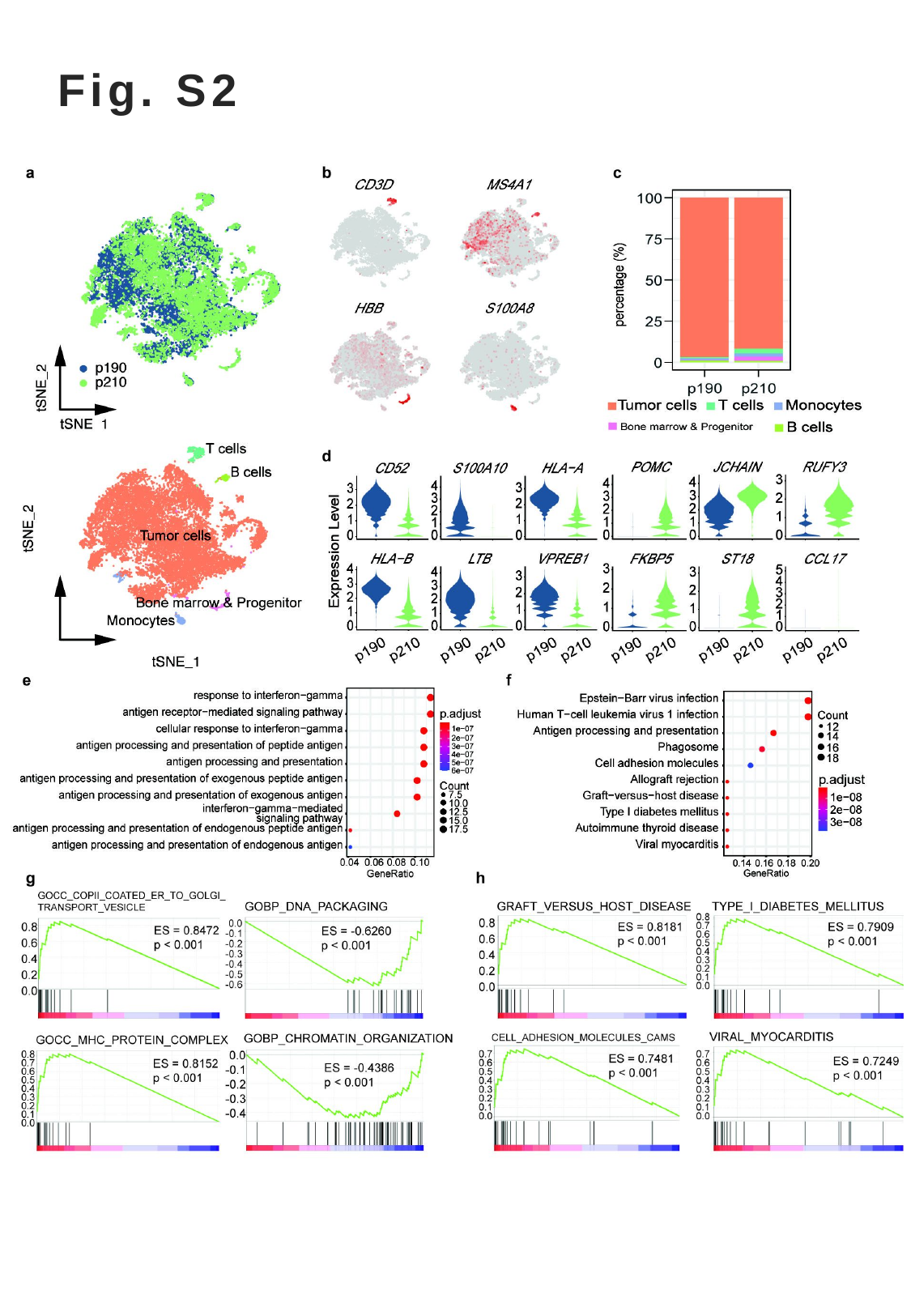

# Fig. S2

Supplement: Supplementary file 1 — Additional file 1: Figure. S1 Outcomes for patients with p210 stratified by treatment protocols (A) Overall survival (OS) and (B) Disease-free survival (DFS). Figure. S2 4 Single-cell RNA-seq analysis. A, tSNE plot of 17,111 cells, colored by the origin samples (top) and cell types (bottom). B, Expression of marker genes for the normal cell types defined above each panel. C, Barplot of ratios of cells in each cell type. D, Violin plots of top 6 upregulated and downregulated differentially expressed genes between two samples among Tumor cells. E, Dotplot of top 10 enriched GO terms of Tumor cells. Enrichment analysis used significantly differentially expressed genes (|log2FC| > 1.5, p value < 0.05 ) between two samples. F, Dotplot of top 10 enriched KEGG pathways of Tumor cells. Enrichment analysis used significantly differentially expressed genes (|log2FC| > 1.5, p value < 0.05 ) between two samples. G, Differences in GO scored by GSEA between b190 and b210 samples’ Tumor cells. H, Differences in KEGG pathways scored by GSEA between b190 and b210 samples’ Tumor cells. [file 40164_2022_265_MOESM1_ESM.pptx]
