# Supplementary material for: Distinct outcomes, ABL1 mutation profile, and transcriptome features between p190 and p210 transcripts in adult Philadelphia-positive acute lymphoblastic leukemia in the TKI era
Source: Exp Hematol Oncol. 2022 Mar 11;11:13. doi: 10.1186/s40164-022-00265-2 (PMC8915539; doi:10.1186/s40164-022-00265-2)
Supplement: Supplementary file 2 — Additional file 2: Table S1. Characteristics of patients included in for relapse and survival analysis. Table S2. Risk Factors for Disease-free survival (DFS) in Univariate and Multivariate Analysis [file 40164_2022_265_MOESM2_ESM.docx]

**Additional file 2: Table S1.**

| **Characteristics** | **p190 (n=143)** | **p210 (n=77)** | ***P*-value** |
| --- | --- | --- | --- |
| **Sex (n,M/F)** | 70/73 | 30/47 | 0.156 |
| **Age [ys,M(range)]** | 42.0 (14.0-76.0) | 44.0 (14.0-71.0) | 0.256 |
| **WBC [*10E9/L,M(range)]** | 19.4 (0.5-403.4) | 32.3 (1.0-403.6) | 0.207 |
| **Hb [g/L,$\overline{x}$±s]** | 100.7±29.1 | 94.1±29.1 | 0.111 |
| **PLT [*10E9/L,M(range)]** | 33.0 (2.0-460) | 40.0 (2.0-403.0) | 0.191 |
| **LDH [U/L,M(range)]** | 621.0 (158.0-7638.0) | 530.0 (153.0-2908.0) | 0.302 |
| **Ferritin [ng/ml,M(range)]** | 872.4 (37.3-88150.0) | 862.9 (13.7-10943.1) | 0.684 |
| **BM blast [%,M(range)]** | 86.0 (23.0-98.0) | 80.5 (22.0-96.0) | 0.007 |
| **Relative BCR/ABL quantification [%,M(range)]** | 43.0(8.3-165.9) | 74.6 (25.0-195.0) | <0.001 |
| **Karyotype (n)**  **With ACAs**  **Without ACAs**  **Unknown** |  |  | 0.459 |
|  | 38 | 19 |  |
|  | 70 | 45 |  |
|  | 35 | 13 |  |
| **CNS diseases (n)** | 12 | 8 | 0.623 |
| **Treatment (n)** | 70  73 | 39  38 | 0.810 |
| **Chemo** |  |  |  |
| **Chemo+Allo-HSCT** |  |  |  |

**Additional file 2: Table S2.**

| **Variable** | **Univariate Analysis** | | | **Multivariate Analysis** | | |
| --- | --- | --- | --- | --- | --- | --- |
|  | **HR** | **95% CI** | ***P*-value** | **HR** | **95% CI** | ***P*-value** |
| **Age [≥35/<35 (ys)]** | 1.188 | 0.663-2.218 | 0.563 | - | - | - |
| **WBC count [≥30/<30 (*10E9/L)]** | 1.367 | 0.852-2.193 | 0.195 | 1.336 | 0.832-2.145 | 0.231 |
| **PLT count [<30/≥30 (*10E9/L)]** | 1.127 | 0.618-1.819 | 0.625 | - | - | - |
| **Cytogenetics (with ACAs/Without ACAs)** | 1.121 | 0.655-1.919 | 0.616 | - | - | - |
| **BCR-ABL1 transcript (p210/p190)** | 1.569 | 0.960-2.565 | 0.073 | 1.543 | 0.942-2.525 | 0.085 |
| **Initial TKIs administrating (2nd/1st)** | 0.880 | 0.480-1.612 | 0.678 | - | - | - |
| **CNS involvement (yes/no)** | 0.843 | 0.362-1.961 | 0.691 | - | - | - |
